# A Novel Image Processing Approach for Colloid Detection in Saturated Porous Media

**DOI:** 10.3390/s24165180

**Published:** 2024-08-10

**Authors:** Behzad Mirzaei, Hossein Nezamabadi-pour, Amir Raoof, Vahid Nikpeyman, Enno de Vries, Reza Derakhshani

**Affiliations:** 1 Intelligent Data Processing Laboratory (IDPL), Department of Electrical Engineering, Shahid Bahonar University of Kerman, Kerman 76169-13439, Iran; 2 Department of Earth Sciences, Utrecht University, 3584 CB Utrecht, The Netherlands; 3 Department of Geology, Shahid Bahonar University of Kerman, Kerman 76169-13439, Iran

**Keywords:** colloid, particle, detection, porous media, image processing, ensemble

## Abstract

Over recent decades, natural and artificial colloids, as well as nanoparticles, have been increasingly used in various applications. Consequently, with this rising consumption, surface and subsurface environments are more exposed to these particles. The presence of these particles and the colloid-facilitated transport of microorganisms, the interactions between dissolved contaminants and mobile colloids in porous media, and the fate and transport of colloids through groundwater—one of the primary sources of water supply for human societies—have attracted extensive research. This study investigates the performance of several image processing methods in the field of colloid detection, which is a prerequisite for the subsequent steps in porous media research. We employed four different categories of image processing approaches on microscopy images—segmentation-based methods, background-detection-based methods, filter-based methods, and morphology-based methods—to conduct the detection process of colloids. Eight methods were applied and subsequently analyzed in terms of their drawbacks and advantages to determine the best ones in this domain. Finally, we proposed an ensemble approach that leverages the strengths of the three best methods using a majority vote to detect colloids more accurately. In experiments, Precision, Recall, *F*-measure, and TCR criteria were considered as evaluation tools. Experimental results demonstrate the high accuracy of image processing methods in recognizing colloids. Among all these methods, morphology-based methods were the most successful, achieving the best detection performance and improving the limited distinguishing features of small colloids. Moreover, our ensemble approach, achieving perfect scores across all evaluation criteria, highlights its superiority compared with other detection methods.

## 1. Introduction

Colloids can be studied at numerous scales, ranging from the pore scale to the kilometer field scale [1,2,3,4,5]. Physical micromodels have proven to be an invaluable tool for studying and observing fluid flow and solute transport within the pore region throughout the last few decades. The highly adjustable physical and chemical conditions of micromodels increase their utility for focusing on pore-scale experimental conditions of interest [5,6,7,8]. They are increasingly being utilized to investigate a variety of topics in reservoir engineering and two-phase flow experiments to identify important mechanisms, such as the effect of interfaces. Micromodels constructed from various materials, including glass, silica, and polydimethylsiloxane (PDMS), were utilized to depict porous media systems.

Due to technological advancement, micro-models can now be studied in digital image format. However, manual analysis of such microscopic images is laborious and very time-consuming. The automated analysis of these types of images to provide quantitative measurements has received considerable attention and has significantly contributed to the advancement of computer-assisted diagnostic systems. Colloid particle identification and tracking are one of the most important tasks in a porous media image analysis.

The goal of this paper is to detect colloids using image processing methods owing to their simplicity, high speed, and accuracy. By doing so, colloids are detected automatically, without the need for manual analysis and with high speed. In previous studies, single methods such as the Laplacian of Gaussian particle detection (LoG detector) were primarily used to detect particles [5,6]. In this study, we select several image processing methods to analyze and compare their results to determine the methods with the best performance for the detection of colloids in our experimental data. Moreover, a novel ensemble detection approach is presented such that it utilizes the detection results of the three best-performing methods to enhance the efficiency of the colloid detection process. Initially, we divide some of the image processing methods into four main groups: segmentation-based methods, background-detection-based methods, filter-based methods, and morphology-based methods. Segmentation-based methods aim to partition an image into meaningful and visually distinct regions or objects, whereas background-detection-based methods are used to detect changes or motion in a video sequence. In filter-based methods, a mask or kernel is utilized to apply to an image and produce the output pixel values. Additionally, morphology-based methods analyze the structure or shape of objects within an image.

From each group, several methods are selected to apply to microscopic images and detect colloids in porous media. We select eight methods to analyze their results and compare them against each other based on various evaluation metrics. Afterward, those methods that have high accuracy in detecting colloids are determined. Finally, we propose an effective ensemble approach to detect colloids in porous media. To do so, the detection results of the three best methods are considered, and colloids are detected based on the majority vote among the obtained results by each method. Thus, we can benefit from the best results of each method and make the detection process more accurate. Concisely, the main contributions of this paper are as follows:

- To detect colloids in porous media, we apply image processing techniques to microscopic images in four different groups and compare their results, which has never been done before.
- The best methods in the field of colloid detection are introduced based on various experiments and several evaluation criteria.
- We propose an ensemble approach to perform the detection process of colloids more effectively through majority voting.
- The proposed methods can detect colloids with high accuracy, despite their simplicity and high speed.

The rest of this paper is organized as follows: Section 2 is dedicated to the experimental setup. In Section 3, we present the proposed methods for detecting colloids in porous media. Section 4 presents the methods and materials used in experiments. A discussion about the obtained results on several evaluation criteria is presented in Section 5. Finally, a summary and conclusions are provided in Section 6.

## 2. Experimental Setup

### 2.1. Solution and Particles

In this study, a fully saturated micromodel (i.e., filled with one solution phase) was used to perform colloid transport. The micromodel included real complex grain and pore geometries. We carried out experiments with a solution having a pH of 6, which is within the normal range for groundwater systems. We used Fluoresbrite YG carboxylate microspheres (Polysciences, Inc.) (Warrington, PA, USA) with a diameter of 4.3 µm as colloidal particles. These types of particles used in our study have a diameter coefficient of variation (CV) of 7%, indicating a relatively narrow size distribution. In the neutral pH range, these colloids are hydrophilic and weakly negatively charged. HPTS, a water-soluble pyrene dye, was used to create a highly fluorescent aqueous solution.

### 2.2. Porous Media

To create micromodels with realistic pore geometries that mimic nature, data from a 3D X-ray tomography on a sand sample were used [5]. A 2D slice was extracted from the 3D image and used to make a mask for the fabrication of a micromodel out of polydimethylsiloxane (PDMS). The pore structures were built using soft lithography methods.

**Figure 1:**
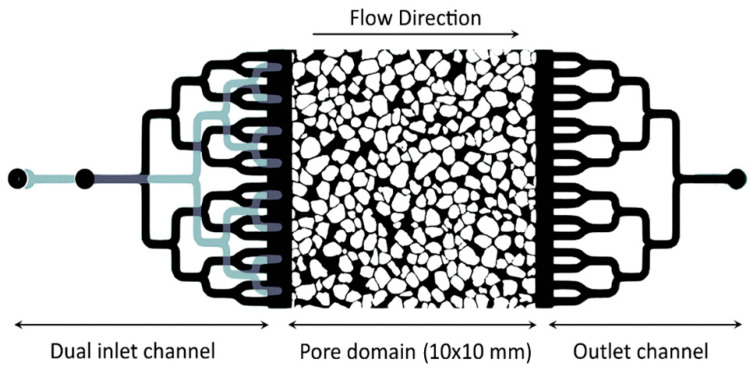
Micromodel structure. The micromodel used in the experiments showing the dual inlet channels for injecting fluid and colloidal suspension; the main pore domain with an area of 10 × 10 mm^2^, a porosity of 0.42, and a depth of 20 µm; and the outlet channels.

In this study, to provide precise control over the injection of colloids into the pore structure, we designed micromodels with two different inlet layers, which were precisely aligned together during the soft lithography process. In Figure 1, two layers are separated using black and gray colors. As illustrated in this figure, the micromodel’s design consists of three main components: (1) inlet channels with 16 branches in the top layer and 8 branches in the bottom layer in order to evenly distribute fluid over the inlet face of the sample; (2) pore domain with an area of 10 × 10 (mm^2^), a porosity of 0.42, and a depth of 20 µm; and (3) output channels.

### 2.3. Experimental Procedure

The experimental system consisted of a series of optical elements and an apparatus to inject the colloids into the micromodel. In order to track the colloids within the pore domain, the micromodel was mounted on a microscope stage (ZEISS Axio Zoom.V16) (Carl Zeiss Microscopy GmbH, Jena, Germany). A dual-drive system syringe pump (Harvard Apparatus Pump 33 DDS) connected through a tube to the micromodel’s main inlet channel was used to create the fluid flow. The micromodel outlet was connected to a vial serving as an exit reservoir. During the experiments, we needed to minimize the disturbances and vibrations of the apparatus to ensure stable imaging. Therefore, the entire procedure was carried out using an optical table system. Throughout the experiment, images with a resolution of 2.9 μm and a frame rate of 10 per second were captured.

The micromodel was initially saturated using the background solution and examined under the microscope to ensure complete saturation. Following that, we injected about 50 pore volumes (PV) of colloid-free water to achieve a fully saturated steady-state flow. The experiment was continued by injecting 4 μL of the solution containing colloids at an average pore water velocity of 4.8 m day^−1^. The average pore water velocity and concentration of colloids employed in this experiment are comparable with the values for pathogenic bacteria and viruses in the subsurface environment of the earth. The colloid injection was then followed by flushing the pore domain with a colloid-free background solution until the mobile colloids were transported outside the pore domain. Therefore, only the attached colloids were still present in the micromodel at the end of the experiment and after being flushed with a background solution.

## 3. The Proposed Detection Methods

**Figure 2:**
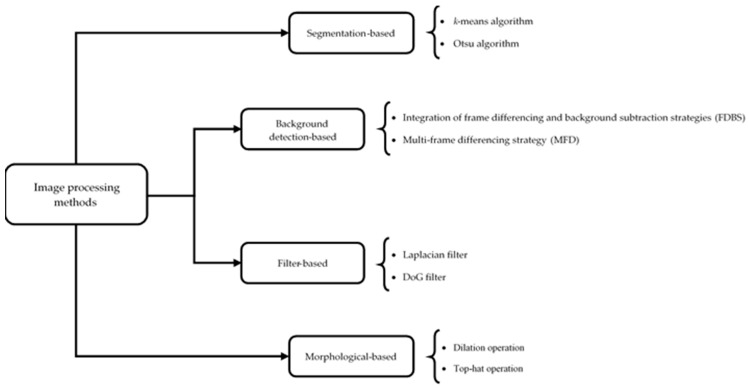
Types of image processing methods. Image processing methods used for colloid detection in porous media, categorized into segmentation-based, background-detection-based, filter-based, and morphology-based methods.

In this section, we present our detection methods. As was mentioned, we apply several image processing techniques to four main groups to detect colloids easily, quickly, and with high accuracy in porous media. Figure 2 shows this taxonomy along with the existing methods in each category. Afterward, based on the best results obtained by three different detection methods, an ensemble approach will be applied to detect colloids using majority voting with more efficiency.

In the following sections, these methods are described in detail:

### 3.1. Segmentation-Based Methods

The division of a digital image into various image segments, often referred to as image regions or image objects (sets of pixels), is a process called image segmentation. The aim of segmentation is to simplify and/or transform an image’s representation into something more relevant and understandable. Image segmentation is frequently utilized to identify objects and boundaries (for example, lines and curves) in images. Image segmentation provides certain labels to image pixels so that pixels with the same label have similar properties, including color, intensity, or texture. Regarding the same properties, adjacent regions are notably different from one another. Image segmentation yields either a collection of segments that cover the entire image totally or a collection of contours that are taken from the image [9,10]. This method has numerous applications in many different fields, such as medical imaging [11], object detection [12], and traffic control systems [13]. There are several types of image segmentation methods used in computer vision and image processing, and some of them are thresholding, region-based, edge detection, clustering-based, and watershed.

In this paper, we use two well-known image segmentation methods in our work, including the *k*-means clustering method [14] and the Otsu method [15], which belong to thresholding and clustering-based categories, respectively. These methods are described as follows:

#### 3.1.1. *k*-Means Clustering

*k*-Means is a clustering technique that divides a set of data points into *k* groups, where *k* is a predetermined integer. Assume that *C* = {$c_{1},\;c_{2},\;\ldots ,\;c_{k}\}$ is the clusters, and *X* = {$x_{1},\;x_{2},\;\ldots ,\;x_{n}\}$ is samples; then the following equations are satisfied:

(1) $$c_{i}\neq \{\}\;\;\;\;\;for\;\;i=1,2,\;\ldots ,\;k$$

(2) $$c_{i}\cap c_{j}=\{\}\;\;\;\;\;for\;\;i,j=1,2,\ldots ,\;k$$

(3) $$\overset{k}{\underset{i=1}{⋃}}c_{i}=X$$

where *n* and *k* denote the number of samples and clusters, respectively. Accordingly, the algorithm works with the following steps [16,17]:

1. *k* samples are selected at random from the set *X* = {$x_{1},\;x_{2},\;\ldots ,\;x_{n}\}$ to be considered as the clusters’ initial centers. We take into account the cluster centers as $z_{1},\;z_{2},\;\ldots ,\;z_{k}$.
2. Each sample in *X* is placed in a cluster that is nearer to its center as follows:
  (4) $$x_{i}\epsilon C_{j}\;\;\;\;\;i=1,2,\ldots ,\;n\;,\;\;j=1,2,\;\ldots ,\;k\;\;\;\;\;\;\;iff\;\;\;\;\;\;{\left\|x_{i},z_{j}\right\|}_{2}<{\left\|x_{i},z_{t}\right\|}_{2}\;\;\;\;t=1,2,\ldots ,k\;\left(j\neq t\;\right)$$
  where the term “if and only if” is denoted by iff and the Euclidean distance is represented by ${\left\|.\right\|}_{2}$.
3. The new cluster centers are calculated using the following equation, if $z_{1}^{\prime },z_{2}^{\prime },\ldots ,z_{k}^{\prime }$ signify them:
  (5) $$z_{j}^{\prime }=\frac{1}{{n\;}_{j}}\sum_{x_{i}\epsilon C_{j}}x_{i}\;\;\;\;\;\;\;\;\;\;\;i=1,2,\ldots ,n_{j}\;,\;\;j=1,2,\ldots ,\;k\;\;$$
  where the number of samples in the cluster $C_{j}$ is represented by the symbol $n_{j}$.
4. If $\left|z_{i}^{\prime }-z_{i}\right|<\varepsilon \;$ for *i* = 1, 2, …, *k*, then the algorithm terminates; otherwise, it loops back to step 2.

The algorithm tries to minimize the within-cluster sum of squares, which is the sum of the squared distances of each data point to its cluster center. It should be mentioned that the initial centers of the clusters have a significant impact on the *k*-means algorithm’s performance, and it may converge to a local optimum. This can be prevented by selecting the initial centers of the clusters properly [16,17,18,19,20].

Using *k*-means clustering, image segmentation can be performed by taking each pixel as a data point and categorizing it into one of the *k* clusters based on its color or intensity value. As a result, each image is divided, and each pixel is given a label that corresponds to its cluster. We apply the *k*-means algorithm to each of the microscopic images to cluster their pixels into different clusters and then detect colloids. We determine the number of clusters (*k*) for the *k*-means algorithm based on the number of regions in each microscopic image. After clustering, there are *k* (*k* > 1) intensity values in the image, one of which is related to colloids such that all of the colloids are placed in a cluster according to their intensity values. Thus, colloids can be separated from other regions of the image.

#### 3.1.2. Otsu Method

The Otsu method is named after Nobuyuki Otsu, who first presented it in 1979 [15]. It is a technique for image thresholding that divides an image into foreground and background areas depending on the grayscale values of its pixels. The goal of Otsu’s approach is to identify an ideal threshold value that maximizes the inter-class variance or, equivalently, minimizes the intra-class variance, which is a metric of how effectively the threshold divides the two areas [21,22,23]. The steps of the Otsu method are as follows:

1. Create the grayscale histogram *H* for image *I* by counting the number of pixels at each intensity level *i* as the following formula:
  (6) $$H(i)=\sum_{x=1}^{M}\sum_{y=1}^{N}\left[I\left(x,y\right)=i\right]$$
  where *M* and *N* stand for the image’s width and height, respectively.
2. Calculate the Cumulative Distribution Function (CDF) as follows:
  (7) $$C(i)=\sum_{j=1}^{i}\frac{H(j)}{M\times N}$$
3. Calculate the mean grayscale intensity value of the image to obtain the inter-class variance as follows:
  (8) $$\mu =\frac{1}{M\times N}\sum_{x=1}^{M}\sum_{y=1}^{N}I(x,y)$$
  where μ is the image’s mean grayscale intensity value.
4. Calculate the inter-class variance for each possible threshold value. The mathematical formulation for the threshold value *T* is as follows:
  (9) $$Var\;\left(T\right)=P_{0}\left(T\right)\times P_{1}\left(T\right)\times {\left(m_{0}\left(T\right)-m_{1}\left(T\right)\right)}^{2}$$
  where *Var* (*T*) denotes the inter-class variance for a given threshold value *T*, and $P_{0}\left(T\right)$ and $P_{1}\left(T\right)$ express the probabilities of the background and foreground areas, respectively. There are the following equations:
  (10) $$P_{0}\left(T\right)=C(T)$$
  (11) $$P_{1}\left(T\right)=1-C(T)$$
  Additionally, the mean grayscale intensity values of the background and foreground regions are specified as $m_{0}\left(T\right)$ and $m_{1}\left(T\right)$. These values can be computed as follows:
  (12) $$m_{0}\left(T\right)=\frac{\sum_{i=0}^{T-1}i\times H(i)}{P_{0}\left(T\right)\times M\times N}$$
  (13) $$m_{1}\left(T\right)=\frac{\sum_{i=T}^{255}i\times H(i)}{P_{1}\left(T\right)\times M\times N}$$
5. Track down the threshold value that maximizes the inter-class variance based on the following equation:
  (14) $$T_{opt}={argmax}_{T}(Var(T))$$
  where $T_{opt}$ represents the optimal threshold *T*, and ${argmax}_{T}$ implies that the selected *T* value should maximize *Var* (*T*).

Once the optimal threshold value is specified, it can be used to convert the grayscale image into a binary image. Accordingly, those pixels in a binary image that have intensity values higher than the threshold are designated as the foreground area. On the contrary, the background area is given to those pixels whose intensity values are below the threshold. We use this segmentation method in our experiments to convert a microscopic image into a binary one and separate colloids from other regions of the image.

### 3.2. Background-Detection-Based Methods

These methods use two strategies. The first strategy, called frame differencing, uses subtracting adjacent frames in the video sequence to detect objects. This strategy considers change in the correlation between the frames of the video sequence and position objects. Meanwhile, the second strategy, known as background subtraction, generating a background model by aligning the frames and subtracting it from each frame, detects existing objects in a video. This strategy is better suited for situations where the camera is fixed, and the background is relatively fixed. Methods that use these two strategies, due to their simplicity and good performance in real-time applications, have attracted much attention [24,25].

One of the most famous methods in this category is two-frame differencing, which can be found in the following formula:

(15) $$d_{n}(x,y)=\left\{\begin{array}{c} 255\;\;\;\;\;\;\;\left|f_{n}\left(x,y\right)-f_{n-1}(x,y)\right|>T\\ \;0\;\;\;\;\;\;\;\;\;\;\;\;\;\;\;\;\;\;\;\;\;\;\;\;\;\;\;\;\;\;\;\;\;\;\;\;\;\;\;\;\;\;\;\;\;\;\;\;\;\;else \end{array}\right.$$

where $d_{n}(x,y)$ refers to the difference frame from two successive frames, *n* is the current frame, (*x*, *y*) indicates the pixel location in the current frame, and $f_{n}\left(x,y\right)$ shows the value of a pixel in the location of the current frame (*x*, *y*). Noise can be eliminated by using the motion threshold value *T*. In the frame differencing methods, the threshold value is crucial for the detection performance. The threshold value, which is always a fixed value, influences the algorithm’s sensitivity in part. A high threshold setting will reduce more noise, but it may also cause a target continuity issue. On the contrary, a low value will cause the final image to retain more noise. A candidate target is considered if the absolute value of the luminance difference exceeds the threshold value [26,27].

The extension of two-frame differencing is the popular three-frame differencing algorithm [24,25]. This algorithm uses a logical “AND” operation to merge two differenced images and considers the result as a foreground image. The following equations denote these principles:

(16) $$d_{1}(x,y)=\left\{\begin{array}{c} 255\;\;\;\;\;\;\;\left|f_{n}\left(x,y\right)-f_{n-1}(x,y)\right|>T\\ 0\;\;\;\;\;\;\;\left|f_{n}\left(x,y\right)-f_{n-1}(x,y)\right|\leq T \end{array}\right.$$

(17) $$d_{2}(x,y)=\left\{\begin{array}{c} 255\;\;\;\;\;\;\;\left|f_{n+1}\left(x,y\right)-f_{n}(x,y)\right|>T\\ 0\;\;\;\;\;\;\;\left|f_{n+1}\left(x,y\right)-f_{n}(x,y)\right|\leq T \end{array}\right.$$

(18) $$d(x,y)=\left\{\begin{array}{c} 255\;\;\;\;\;\;\;if\;\;\;\;d_{1}\left(x,y\right)=255\;\;\;\;and\;\;\;\;d_{2}\left(x,y\right)=255\\ \;\;0\;\;\;\;\;\;\;\;\;\;\;\;\;\;\;\;\;\;\;\;\;\;\;\;\;\;\;\;\;\;\;\;\;\;\;\;\;\;\;\;\;\;\;\;\;\;\;\;\;\;\;\;\;\;\;\;\;\;\;\;\;\;\;\;\;\;\;\;\;\;\;\;\;else \end{array}\right.$$

In this paper, we use two different methods from this category to detect colloids, which are described as follows:

#### 3.2.1. Frame Differencing Plus Background Subtraction (FDBS)

This method integrates frame differencing and background subtraction strategies to detect objects [28]. If the current frame is *A*, and the previous frame is *B*, small colloids from *A* can be detected as follows:

1. The frame difference between *A* and *B* is carried out, and the result is expressed as *C*.
2. A background subtraction of frame *A* with the background model is performed, and the result is expressed as *D*.
3. The logical AND operation between *C* and *D* is performed to obtain a result frame containing detected colloids.

**Figure 3:**
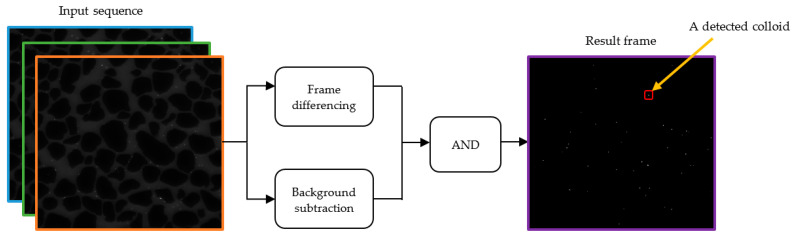
Frame differencing plus background subtraction (FDBS) method. The FDBS method for detecting colloids, integrating frame differencing and background subtraction strategies.

Figure 3 depicts this detection method for our work.

#### 3.2.2. Multi-Frame Differencing (MFD)

We use another algorithm for the detection process, which uses a multi-frame differencing strategy. This algorithm first selects three consecutive frames from the video sequence as $f_{t-1}$, $f_{t}$, $f_{t+1}$. In the next step, the differences between these frames are computed as the following equations:

(19) $$d_{t1}=\left|f_{t}-f_{t-1}\right|$$

(20) $$d_{t2}=\left|f_{t+1}-f_{t-1}\right|$$

(21) $$d_{t3}=\left|f_{t+1}-f_{t}\right|$$

where $d_{t1}$, $d_{t2}$, and $d_{t3}$ denote the difference results. Then, the average of these differences is obtained as follows:

(22) $$f_{d}=\frac{d_{t1}+d_{t2}+d_{t3}}{3}$$

After the response image $f_{d}$ is achieved, the binarization is conducted on this image [29]. To do so, the pixels that have values greater than the threshold *T* are retained, while the rest are set to zero, as follows:

(23) $$f_{d}=\left\{\begin{array}{c} 255\;\;\;\;\;\;f_{d}(x,y)>T\\ 0\;{\;\;\;\;\;\;\;\;\;f}_{d}(x,y)\leq T \end{array}\right.$$

**Figure 4:**
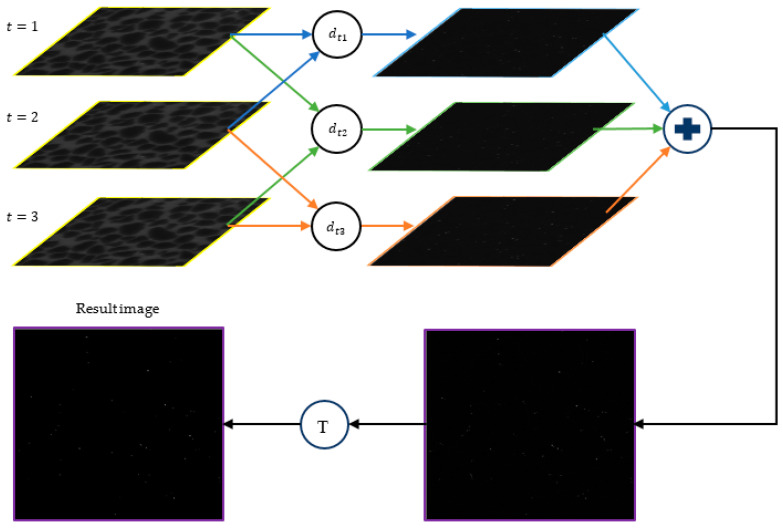
Multi-frame differencing (MFD) method. The MFD method for colloid detection, involving the calculation of differences between three consecutive frames and averaging these differences.

Figure 4 shows the procedure for this method for the detection of colloids. Generally, background-detection-based methods have the advantages of simplicity, low computational complexity, and high speed. However, these methods have drawbacks with producing a lot of noise as the target object. Therefore, using post-processing techniques to eliminate noise after detection is crucial. On the other hand, it is also possible that they miss some objects such that non-moving objects and objects that move slowly will not be detected by these methods [27,30,31,32,33,34].

### 3.3. Filter-Based Methods

These techniques use a mask or a kernel to process an image and generate the output pixel values from the input pixel values and the surrounding pixels. They can be either linear or nonlinear methods. Spatial filters that have a linear response to all the gray values in the mask are called linear filters. This means that the output pixel is the result of a dot product of the pixel values and the mask coefficients in the neighborhood of the original pixel in the image. Spatial filters that choose the output pixel from an ordered sequence of pixel values around the original pixel in the image are known as nonlinear filters. In this study, we use linear filters, specifically the Laplacian and Difference of Gaussians (DoG) filters, to detect small colloids in microscopic images.

#### 3.3.1. Laplacian Filter

The Laplacian filter is a second-order or a second-derivative technique of enhancement that emphasizes areas of fast intensity variation in an image. It is very effective at revealing the fine details of an image. Any feature that has a sharp discontinuity is enhanced by this filter [35].

Laplacian is a famous linear differential operator that estimates the second derivative, as shown in the following Equation (24):

(24) $$\nabla^{2}f=\frac{\partial^{2}f}{\partial x^{2}}+\frac{\partial^{2}f}{\partial y^{2}}$$

where *f* represents the image.

This filter is a high-pass filter that responds to sharp changes in the intensity of the image and shows points with higher or lower intensity around it. We use this Laplacian property to detect colloids that have higher brightness than the background. If there is a single-pixel bright object like a colloid in a dark background, the Laplacian filter will create a white dot at the location of the object and four black dots around it. Therefore, by searching for such a dot pattern in the output of the Laplacian filter, we can find the location of the colloid.

#### 3.3.2. Difference of Gaussians (DoG) Filters

Difference of Gaussians (DoG) is the result of subtracting two smoothed versions of an image that are created by applying two Gaussian kernels with different sigma (standard deviation) values to that image. This means that the DoG operation on an image involves taking away one very smoothed version of the original image from another slightly smoothed version to work as a band-pass filter that keeps a certain spatial frequency [36,37]. If the 2D Gaussian function is defined as follows:

(25) $$G\left(x,y,\sigma \right)=\frac{1}{2\pi \sigma^{2}}exp(-\frac{x^{2}+y^{2}}{2\sigma^{2}})$$

where σ denotes the standard deviation of the Gaussian, then the difference of two Gaussians with different sigma values creates a DOG filter, as shown in the following Equation (26):

(26) $$G\left(x,y,\sigma_{1},\sigma_{2}\right)=G\left(x,y,\sigma_{1}\right)-G\left(x,y,\sigma_{2}\right)=\frac{1}{2\pi }\left(\frac{1}{\sigma_{1}^{2}}\exp\left(-\frac{x^{2}+y^{2}}{2\sigma_{1}^{2}}\right)-\frac{1}{\sigma_{2}^{2}}\exp\left(-\frac{x^{2}+y^{2}}{2\sigma_{2}^{2}}\right)\right)$$

where $\sigma_{1}<\sigma_{2}$. This filter is a spatial band-pass filter that attenuates frequencies far from the band center and highlights points that have sharp changes in intensity. These points are usually indicative of sharp edges or image corners. Hence, to detect colloids that have the highest intensity in a microscopic image, we consider the zero-crossing points of the DoG filter where there is an extreme change in brightness.

### 3.4. Morphology-Based Methods

Morphology is a wide range of image processing operations that process images depending on shapes. Morphological operations by applying a structural element to an input image produce an output image of the same size. The structuring element establishes the area that will be examined surrounding each pixel. In a morphological operation, the value of each pixel in the output image is determined by comparing it with its neighbors in the corresponding pixel in the input image. According to features of the image’s shape that are encoded in the structuring element, the objects in the input image are processed. In binary morphology, an image is seen as a subset of a Euclidean space $R^{d}$ or the integer grid $Z^{d}$ for some dimension *d* [38,39].

#### 3.4.1. Structuring Element

Binary morphology’s fundamental idea is to probe an image with a straightforward, pre-defined shape and make judgements about how well or poorly it matches the shapes in the image. The structuring element is a straightforward “probe” that is itself a binary image [38,39]. Examples of frequently used structural elements are shown below (expressed by *B*):

1. Let $E=R^{2}$; *B* is an open disk with a radius of r and origin-centered.
2. Let $E=Z^{2}$; *B* is a 3-by-3 square; thus, *B* = {(−1, −1), (−1, 0), (−1, 1), (0, −1), (0, 0), (0, 1), (1, −1), (1, 0), (1, 1)}.
3. Let $E=Z^{2}$; *B* is the “cross” indicated by *B* = {(−1, 0), (0, −1), (0, 0), (0, 1), (1, 0)}.

#### 3.4.2. Basic Operations

There are several basic morphological operations for binary images, such as erosion, dilation, top-hat, opening, and closing. These operations can be used to recognize features of interest in images, improve feature contrast, or prepare images for subsequent analysis [38,40,41,42]. Here, some popular operations are described as follows:

❖ Erosion

Erosion is a morphological procedure that reduces and thins out the boundaries of objects in an image by removing pixels. Assume that *A* is a binary image in *E* and that *E* is either an integer grid or a Euclidean space; then the erosion of the binary image *A* by the structuring element *B* can be conducted as follows:

(27) $$A⊖B=\left\{z\in E|B_{z}\subseteq A\right\}$$

where $B_{z}$ denotes the translation of *B* by the vector *z,* that is,

(28) $$B_{z}=\left\{b+z|b\in B\right\},\forall z\in E$$

❖ Dilation

Dilation enlarges and improves the visibility of image objects by adding pixels to their borders. The dilation of *A* by the structuring element *B* is performed as the following equation:

(29) $$A⨁B=\left\{z\in E|{\left(B^{s}\right)}_{z}\cap A\neq \emptyset \right\}$$

where $B^{s}$ indicates the symmetric of *B*, i.e.,

(30) $$B^{s}=\left\{x\in E|-x\in B\right\}$$

❖ Opening

The opening in morphology is a type of image processing operation that involves erosion followed by dilation and is defined as follows:

(31) A∘B=(A⊖B)⊕B

By applying opening, the image becomes smoother and the small bright regions or noise is eliminated. The opening can also help identify shapes that fit a specific structuring element.

❖ Top-hat

The top-hat operation is also known as the white top-hat transform ${(T}_{w})$, because it preserves the bright regions that are smaller than the structuring element. This operation is obtained based on the difference between the original image and its opening by the structuring element as follows:

(32) $$T_{w}(A)=A-(A\circ B)$$

The bright regions eliminated by the opening operation are the only ones retained by the top-hat operation. These regions are usually brighter than their surroundings and are small details or noise.

In this study, we apply dilation and top-hat operations to our microscopic images to evaluate their ability to detect small colloids in porous media. Dilation can expand or enlarge the bright regions in an image. Therefore, we can increase the size of small colloids in images and, as a result, enhance their features using this operation, which leads to better detecting them. On the other hand, using the top-hat operation, we can extract small objects and details such as small colloids from the given images.

### 3.5. Ensemble Approach

**Figure 5:**
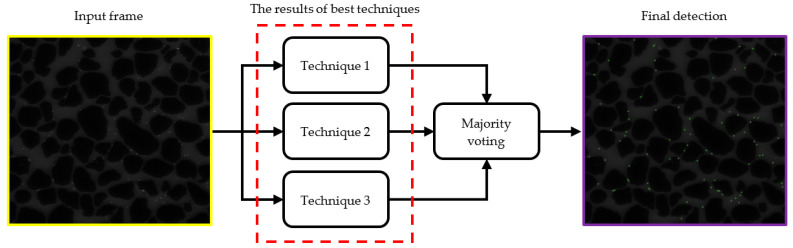
Ensemble approach for colloid detection. Block diagram of the proposed ensemble approach for colloid detection, combining the results of the three best-performing methods using majority voting.

After applying the abovementioned eight image processing techniques to microscopic images and performing the detection process of colloids, we suggest an ensemble approach to take advantage of the best of them. To do so, among the results of three image processing techniques with the best detection performance, a majority vote is taken to make the final decision whether to detect or not to detect a colloid. For example, if two methods decided to detect a colloid and the decision of the other method was not to detect it, the final decision of the ensemble approach would be to detect that colloid. Figure 5 illustrates a block diagram of this ensemble approach for the detection of colloids.

## 4. Methods and Materials

**Figure 6:**
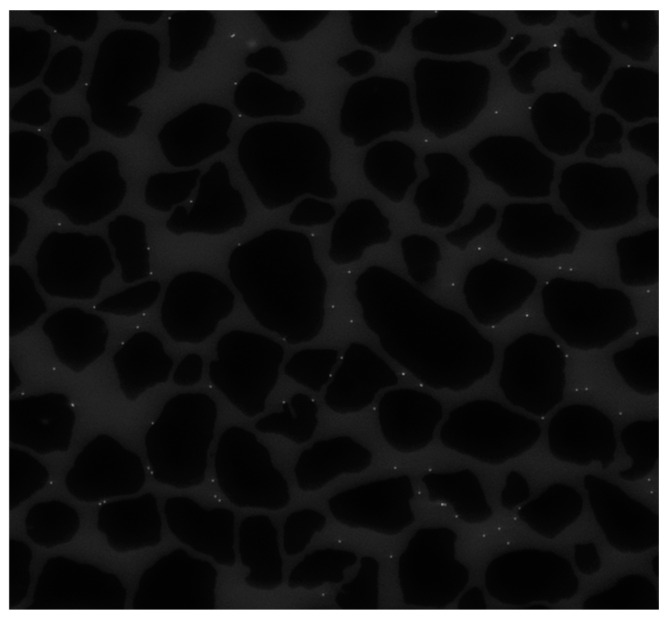
Grayscale image of micromodel. Grayscale image of the micromodel used in the experiments, showing solid grains (in black), pore space (in light gray), and colloidal particles (in white dots).

We implemented our programs including eight image processing techniques plus an ensemble approach in MATLAB (R2022a) software. The dataset used in this paper comprises 500 microscope images such that the colloid in the images has been recorded and handled in many time frames. All of the images are grayscale images. The images are of the same size, measuring 1684 pixels in height and 1688 pixels in width. We use 25 images from this dataset in our experiments to analyze the performance of detection algorithms. The first frame from this dataset is illustrated in Figure 6, which has 81 colloidal particles in total. It should be noted that we manually generate ground truth for 25 images to identify these particles. Each frame has three regions: solid grains, porous media, and colloids. In Figure 6, black sections show the solid grains, light gray sections show porous media, and white dots represent colloids. Note that solid grains and porous media belong to the background and are static. It is just the colloids that move during the video frames in porous media. Moreover, the number of colloids in each of the 25 frames analyzed from the experimental data varies due to the differing velocities and trajectories of individual colloids, along with the continuous entry and exit of colloids from the domain in each frame. Since the colloids used in this study are small particles with a diameter of 4.3 µm, their exact detection is a challenge, because these objects have poor appearance and features to be identified and distinguished from other regions.

### 4.1. Performance Evaluation Metrics

To evaluate the performance of our proposed methods quantitatively, we employ four evaluation metrics: Precision, Recall, *F*-measure, and Target-to-Clutter Ratio (TCR). The following are their detailed definitions:

#### 4.1.1. Precision, Recall, and *F*-Measure

Before defining these metrics, we should state some concepts as follows [16,29,34,43,44]:

- True positive (*TP*): detecting a target correctly;
- False positive (*FP*): detecting a nonexistent target incorrectly, or a misplaced detection of an existing target;
- False negative (*FN*): a target that has not been detected.

The evaluation of the object detection techniques is usually based on the Precision and Recall concepts, which are stated as follows:

(33) $$Precision=\frac{TP}{TP+FP}$$

(34) $$Recall=\frac{TP}{TP+FN}$$

Since there is a trade-off between the Precision and Recall values, a high Recall value will result in a low Precision value and vice versa. The harmonic mean of Precision and Recall, known as *F*-measure, is a traditional criterion for the binary classification of interest targets and non-targets as follows:

(35) $$F-measure=\frac{2\times Precision\times Recall}{Precision+Recall}$$

The higher *F*-measure results from higher Precision and Recall scores, indicating that there is not a significant difference between the two [45].

#### 4.1.2. Target-to-Clutter Ratio (TCR)

We use another comprehensive criterion in our experiments named, TCR, which is defined as follows [46,47]:

(36) $$TCR=\frac{DT}{DT+MT+FA}$$

where, in a frame,

- Detected targets (*DT*) is the number of detected targets;
- Missed targets (*MT*) is the number of missed targets;
- False alarm (*FA*) denotes false alarm detections or incorrect detections.

A score of one for these metrics is the best possible score, and lower values indicate poorer performance.

In this paper, we consider *FA* = *FP* and *MT* = *FN*. Additionally, our targets are colloids. It should be noted that each colloid is detected only once, so FP or FA detections are assigned to any additional detections in the surrounding region. Moreover, for a merged detection, we count one detection as *TP* and assign *FN*s or *MT*s to the rest of colloids in the merged region.

### 4.2. Non-Parametric Statistical Test for Statistical Analysis

The Friedman test [48], a non-parametric statistical method, is used to statistically evaluate the performance of algorithms. This test begins with the assumption that the competing algorithms have equivalent performance (null hypothesis). The main objective is to determine whether the null hypothesis should be rejected or not. If this hypothesis is rejected, it indicates a discrepancy between the algorithms, allowing the acceptance of the alternative hypothesis. This discrepancy is assessed by calculating the *p*-value; if the *p*-value is less than or equal to a specified significance level, the null hypothesis will be rejected. In this paper, the significance level is set at α=0.05. At the end of this step, rankings are calculated to determine which method has the lower rank, thereby determining the controlling method. In the next step, a post hoc procedure can be conducted to specify differences among algorithms. In experiments, we apply Holm’s test as a post hoc procedure to calculate the adjusted *p*-values [49].

## 5. Results and Discussion

**Figure 7:**
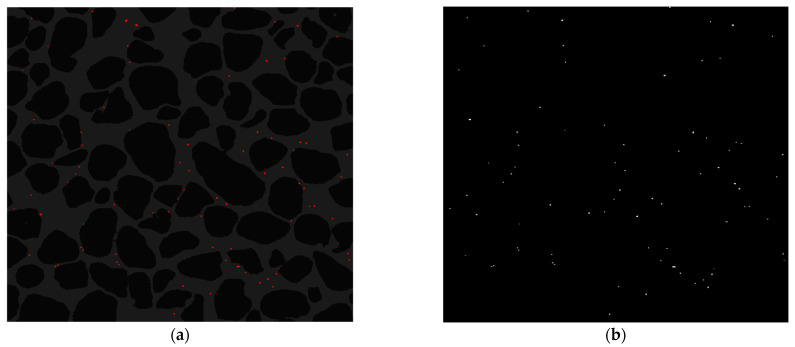
Colloid detection using segmentation-based methods. Results of colloid detection in a frame of experimental data using (**a**) the *k*-means algorithm and (**b**) the Otsu algorithm. Detected colloids are highlighted in red in (**a**) and separated from the background in (**b**).

In this section, the results of our proposed methods are evaluated using several popular evaluation metrics, and their effectiveness in the detection process of colloids is demonstrated. To perform the *k*-means algorithm on microscopic images, the number of clusters is set to 3 due to the presence of three regions in each image: solid grains, porous media, and colloids. This means that all colloids are placed inside a cluster and, therefore, are identifiable. The result of applying the *k*-means algorithm to an image for detecting colloids in porous media is shown in Figure 7a. The detected colloids are highlighted in red. As can be seen, there are three clusters, and colloids have been well detected.

Figure 7b illustrates the obtained result by the Otsu method for the detection of colloids. It can be seen that the given image has been converted into a binary image and has two regions: background and foreground. According to Figure 7b, the foreground region including colloids is easily identifiable. In other words, this method has the ability to separate colloids from the background very well.

**Figure 8:**
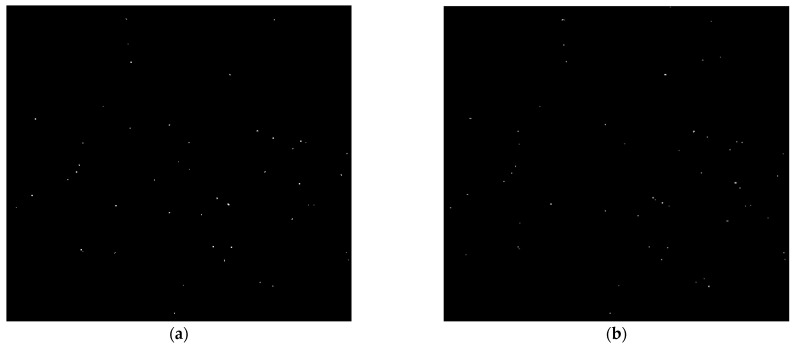
Colloid detection using background-detection-based methods. Results of colloid detection in a frame of experimental data using (**a**) the FDBS method and (**b**) the MFD method. These methods miss some colloids and produce some noise.

Figure 8a,b show the result of colloid detection by background-detection-based methods including FDBS and MFD methods employed in this paper, respectively. According to these results, background-detection-based methods miss some colloids in the detection process, because they are suitable for detecting moving colloids. They cannot detect non-moving colloids and colloids that move slowly due to their nature. Additionally, the results show that these methods are prone to producing some noise after detecting objects like colloids in the images.

**Figure 9:**
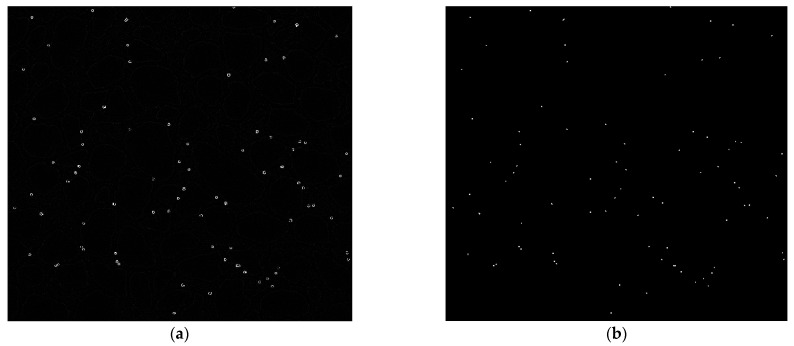
Colloid detection using filter-based methods. Results of colloid detection in a frame of experimental data using (**a**) the Laplacian filter and (**b**) the Difference of Gaussian (DoG) filter. Both methods highlight the boundaries of colloids.

Figure 9a depicts the result of the Laplacian filter for the detection of colloids. A Laplacian filter is a 2D filter that enhances the edges and corners of an image by computing the second derivatives of the image intensity. This filter, by highlighting the regions where there is a rapid change in image intensity, can identify the boundaries of the objects like colloids and separate them from the background, as shown in Figure 9a. As can be seen, most colloids have been successfully detected.

To perform the Difference of Gaussian (DoG) filter on microscopic images, we select $\sigma_{1}=0.5$ and $\sigma_{2}=2$. Additionally, the size of the filter is selected as 3×3. These values are determined through experiments. A DoG filter is a 2D filter that approximates the Laplacian of Gaussian (LoG) filter, which is a combination of a Gaussian smoothing filter and a Laplacian filter. The DoG filter works based on subtracting two Gaussian filters with different standard deviations (sigma). Similar to the Laplacian filter, the DoG filter, by highlighting the regions where there is a significant difference between the neighboring pixels’ intensities, can identify the boundaries of our colloids and separate them from the background. The result of detection by this filter is shown in Figure 9b. This Figure shows that colloids are detected with high accuracy.

**Figure 10:**
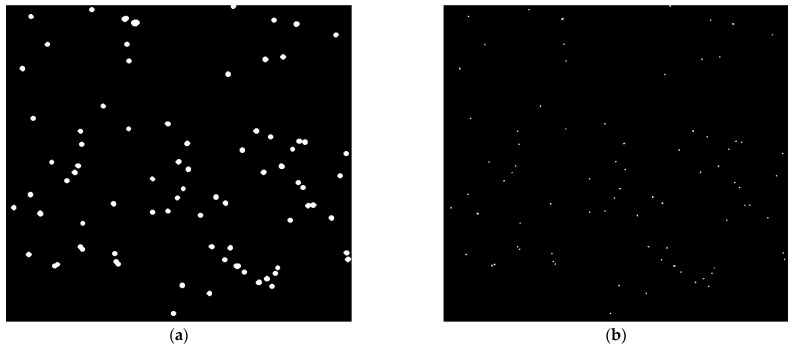
Colloid detection using morphology-based methods. Results of colloid detection in a frame of experimental data using (**a**) the dilation operation and (**b**) the top-hat operation. The dilation operation expands the size of small colloids, while the top-hat operation enhances the contrast between colloids and their surroundings.

Figure 10a,b illustrate the obtained results by morphology-based methods, including dilation and top-hat operations, respectively. It should be noted that the structuring elements used for dilation and top-hat operations are a disk with a radius of four pixels and two pixels, respectively. The dilation operation, by adding pixels to the boundaries of small colloids that occupy only one pixel in the image, expands them. Hence, this operation can increase the size of small colloids and improve their features to better detect them, as shown in Figure 10a. On the other hand, the top-hat operation, by enhancing the contrast between colloids and their surroundings, has high accuracy in their detection process based on Figure 10b.

Table 1 displays the average rankings obtained from the Friedman test applied to the *F*-measure results on 25 different microscopic images for all detection methods. In this table, algorithms are listed based on the order of their average ranks. As can be seen, top-hat achieves the best Friedman ranking with a rank of 2.34. Moreover, DoG and *k*-means methods stand in the second and third places with ranks of 2.42 and 2.50, respectively. Table 2 presents the results of Holm’s test along with the *p*-values obtained for *F*-measure. The very small *p*-values (approaching zero) are represented in scientific notation. These values indicate extremely strong evidence against the null hypothesis. This means that the differences between the compared algorithms are extremely statistically significant. The obtained results confirm the significant superiority of top-hat, DoG, and *k*-means over the other methods by achieving *p*-values lower than α = 0.05. Additionally, these three methods do not outperform each other significantly. As a result, it can be stated that top-hat, DoG, and *k*-means methods, with the best *F*-measure rankings, significantly outperform other detection methods.

The average results of four evaluation criteria, including Precision, Recall, *F*-measure, and TCR on 25 images for each detection method, are reported in Table 3. Additionally, the standard deviation for each evaluation criterion is given in front of the results of each method. This table shows that image processing methods and also our ensemble approach perform well in detecting colloids while being simple and fast. To perform the ensemble approach, we use the results of three detection algorithms comprising top-hat operation, DoG filter, and *k*-means clustering, which have the best performance of detection among the algorithms based on their results. As can be seen, the best results belong to the ensemble approach, which has attained the score of one for all evaluation metrics. This shows that all colloids are correctly detected and there are no incorrect detections and missed colloids in this approach. The DoG filter and the top-hat operation stand in the second and third places, respectively. It should be noted that the better results of *F*-measure achieved by these methods indicate that both Precision and Recall have higher values and there is not much difference between them. By looking at Table 3, it is observed that some methods due to FP = FA = 0 have reached the score of one for Precision and have the same results for Recall and TCR.

Background-detection-based methods show poor performance by Recall, *F*-measure, and TCR, and have the lowest values in terms of these metrics. The major reason is that these methods cannot detect non-moving colloids and colloids that move slowly. Since there are a lot of such colloids in the video sequence, they encounter high missed colloids, which lead to high FN or MT. Moreover, the MFD method used from this category has the least average value of Precision due to generating a lot of noise, which leads to reaching high FP or FA. The DoG filter and the top-hat operation have attained a score of one for the Recall criterion, which means that these methods do not miss any colloid and can detect all of them, but they have some incorrect detections. On the other hand, the proposed ensemble approach has achieved the best results in terms of all evaluation criteria by considering the results of the superior three methods and applying the majority vote between all of them. As Table 3 illustrates, the dilation operation achieves good results as a detection method. In addition, this operation, by improving the poor appearance and limited distinguishing features of small colloids in microscopic images, can help their detection process and the subsequent steps in porous media research. Therefore, this lack of information can be solved using dilation. Generally, it can be concluded that morphology-based methods such as dilation and top-hat operations, due to their good performance, are more suitable than the existing methods in the other three categories in the field of detecting colloids in porous media.

**Figure 11:**
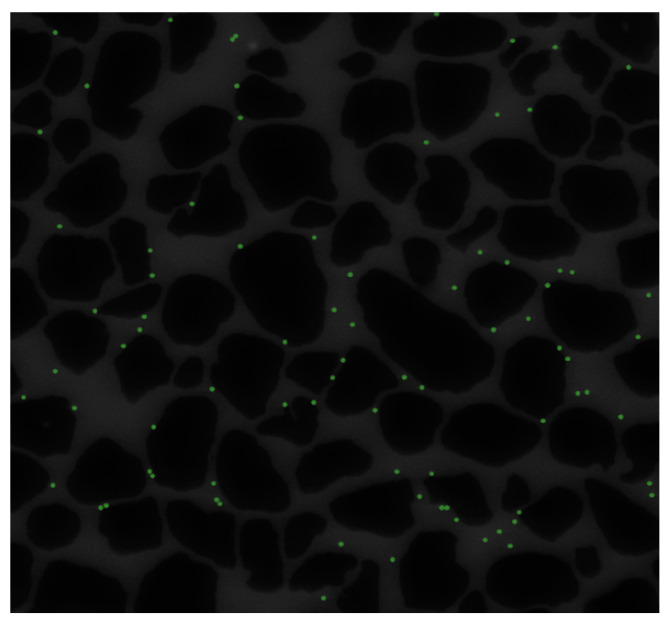
Colloid detection using the ensemble approach. Result of detecting colloids in a frame of experimental data using the proposed ensemble approach. Detected colloids are highlighted in green, demonstrating the effectiveness of the ensemble approach in accurately identifying all colloids in the image.

Eventually, the result achieved by our ensemble approach is presented in Figure 11. The detected colloids are highlighted in green. The ensemble approach can correct the errors of the individual detection methods by combining their outputs and selecting the most common one. As Figure 11 illustrates, this approach, using majority voting among the best results obtained by three different detection methods, is capable of detecting colloids effectively and with a desirable performance. Based on Figure 11, it can be stated that all 81 colloids in the image are correctly identified.

## 6. Summary and Conclusions

This manuscript presents a novel approach for the detection of colloids in fully saturated colloid transport experiments using image processing techniques. We categorized these techniques into four groups: segmentation-based methods, background-detection-based methods, filter-based methods, and morphology-based methods. Subsequently, we applied eight different methods chosen from these groups to investigate their ability and identify the best ones for detecting colloids in porous media. Finally, we proposed an ensemble approach that uses majority voting among the top 3 methods—the top-hat operation, DoG filter, and *k*-means clustering—to identify colloids more accurately. A comprehensive set of experiments under several evaluation metrics was conducted on microscopic images to illustrate the effectiveness of our proposed methods. The experimental results proved that, while being simple and fast, image processing techniques have desirable performance in the detection process of colloids.

The main findings of this study include the following:

- The effectiveness of our ensemble approach was demonstrated, achieving the best results in terms of all evaluation metrics with a perfect score of one.
- After the proposed ensemble approach, the DoG filter and the top-hat operation exhibited the best detection performance on average.
- Background-detection-based methods had the worst results compared with other methods because they cannot detect non-moving colloids and colloids that move slowly. Additionally, these methods produce a lot of noise in the detection process, necessitating post-processing algorithms.
- Since small colloids do not have enough information to identify them, the dilation operation, by expanding the boundaries and increasing the size of small colloids, can improve their distinguishing features for detection and subsequent research on porous media, such as colloid tracking.
- The presented results confirmed that morphology-based methods perform the process of detecting colloids in porous media more effectively and are more useful in this field compared with the methods of the other three categories.

In this work, an image processing approach is utilized for the detection of colloids in porous media. In future work, we are interested in applying artificial intelligence in the field of colloid detection. Furthermore, the dilation operation can be tested to improve the poor appearance and limited distinguishing features of small colloids and enhance the performance of detection methods.

## Figures and Tables

**Table 1:** Average Friedman rankings obtained for *F*-measure.

Types of Methods	Ranking
Top-hat	2.34
DoG	2.42
*k*-Means	2.50
Otsu	3.94
Laplacian	4.44
Dilation	5.36
FDBS	7.20
MFD	7.80

**Table 2:** Post hoc comparison for α=0.05 based on *F*-measure.

I	Methods	*p*-Value	Holm
1	DoG vs. Top-hat	0.908073	0.05
2	*k*-Means vs. DoG	0.908073	0.025
3	*k*-Means vs. Top-hat	0.817361	0.016667
4	Otsu vs. Laplacian	0.470486	0.0125
5	FDBS vs. MDF	0.386476	0.01
6	Laplacian vs. Dilation	0.184209	0.008333
7	Otsu vs. Dilation	0.040404	0.007143
8	*k*-Means vs. Otsu	0.037667	0.00625
9	Otsu vs. DoG	0.02824	0.005556
10	Otsu vs. Top-hat	0.020291	0.005
11	FDBS vs. Dilation	0.007912	0.004545
12	*k*-means vs. Laplacian	0.005108	0.004167
13	Laplacian vs. DoG	0.00355	0.003846
14	Laplacian vs. Top-hat	0.002437	0.003571
15	MDF vs. Dilation	0.000429	0.003333
16	FDBS vs. Laplacian	0.000068	0.003125
17	*k*-Means vs. Dilation	0.000037	0.002941
18	DoG vs. Dilation	0.000022	0.002778
19	Dilation vs. Top-hat	0.000013	0.002632
20	Otsu vs. FDBS	0.000003	0.0025
21	MDF vs. Laplacian	0.000001	0.002381
22	Otsu vs. MDF	<$1.0\times 10^{-6}$	0.002273
23	*k*-Means vs. FDBS	<$1.0\times 10^{-6}$	0.002174
24	FDBS vs. DoG	<$1.0\times 10^{-6}$	0.002083
25	FDBS vs. Top-hat	<$1.0\times 10^{-6}$	0.002
26	*k*-Means vs. MDF	<$1.0\times 10^{-6}$	0.001923
27	MDF vs. DoG	<$1.0\times 10^{-6}$	0.001852
28	MDF vs. Top-hat	<$1.0\times 10^{-6}$	0.001786

**Table 3:** Average results obtained by different detection methods under several evaluation metrics.

Types of Methods		Metrics			
		Precision	Recall	*F*-Measure	TCR
Segmentation-based	*k*-Means	0.9949 (±0.007)	0.9799 (±0.010)	0.9873 (±0.007)	0.9752 (±0.014)
	Otsu	1.0000 (±0.000)	0.9646 (±0.013)	0.9819 (±0.007)	0.9646 (±0.013)
Background-detection-based	FDBS	1.0000 (±0.000)	0.7127 (±0.062)	0.8309 (±0.044)	0.7127 (±0.062)
	MFD	0.8905 (±0.057)	0.7301 (±0.038)	0.8005 (±0.018)	0.6933 (±0.024)
Filter-based	Laplacian	1.0000 (±0.000)	0.9595 (±0.016)	0.9793 (±0.008)	0.9595 (±0.016)
	DoG	0.9754 (±0.008)	1.0000 (±0.000)	0.9875 (±0.004)	0.9761 (±0.008)
Morphology-based	Dilation	0.9843 (±0.006)	0.9543 (±0.026)	0.9689 (±0.015)	0.9411 (±0.029)
	Top-hat	0.9752 (±0.000)	1.0000 (±0.000)	0.9874 (±0.000)	0.9758 (±0.000)
Ensemble		1.0000 (±0.000)	1.0000 (±0.000)	1.0000 (±0.000)	1.0000 (±0.000)

## Data Availability

The datasets generated and analyzed in this study are not publicly available, as they are part of an ongoing Ph.D. project. However, they can be obtained from the corresponding author upon reasonable request.
